# Dynamic Interactive Social Cognition Training in Virtual Reality (DiSCoVR) for social cognition and social functioning in people with a psychotic disorder: study protocol for a multicenter randomized controlled trial

**DOI:** 10.1186/s12888-019-2250-0

**Published:** 2019-09-05

**Authors:** Saskia A. Nijman, Wim Veling, Kirstin Greaves-Lord, Rowina R. Vermeer, Maarten Vos, Catharina E. R. Zandee, Daniëlle C. Zandstra, Chris N. W. Geraets, Gerdina H. M. Pijnenborg

**Affiliations:** 1 Department of Psychotic Disorders, GGZ Drenthe, Dennenweg 9, PO Box 30007, 9404 LA Assen, the Netherlands; 2 University Center of Psychiatry, University Medical Center Groningen, University of Groningen, Hanzeplein 1, PO Box 30.001, 9700 RB Groningen, the Netherlands; 3 Department of Child and Adolescent Psychiatry/Psychology, Erasmus MC-Sophia, Wytemaweg 8, 3015 CN Rotterdam, The Netherlands; 4 Autism Team Northern-Netherlands of Jonx, Department of (Youth) Mental Health and Autism of Lentis Psychiatric Institute, Laan Corpus den Hoorn 102-2, 9728 JR Groningen, The Netherlands; 5 Department of Yulius Autism, Yulius, Amazone 7, 3315 WG Dordrecht, the Netherlands; 6 Flexible Assertive Community Treatment Team, Outpatient Treatment Center, GGZ Delfland, Sint Jorisweg 2, 2612 GA Delft, The Netherlands; 7 Zeeuwse Gronden, Axelsestraat 8/A, 4537 AJ Terneuzen, The Netherlands; 8 Department of Psychology, University of Groningen, Grote Kruisstraat 2/1, 9712 TS Groningen, The Netherlands

**Keywords:** Social cognition training, Virtual reality, Emotion perception, Theory of mind, Psychotic disorder, Social functioning

## Abstract

**Background:**

Problems in social functioning (e.g., unemployment, social isolation), are common in people with a psychotic disorder. Social cognition is a treatment target to improve social functioning, as it is a proximal predictor of social functioning. Social Cognition Training (SCT) improves social cognition, but may not generalize (enduringly) to social functioning, perhaps due to insufficient opportunity to practice in daily-life social situations. Using virtual reality (VR) for SCT could address this problem, as VR is customizable, accessible, and interactive. We will test the effect of a VR SCT, ‘DiSCoVR’, on social cognition and social functioning in a randomized controlled trial (RCT).

**Methods:**

In total 100 people with a psychotic disorder and deficits in social cognition will be recruited for this multicenter randomized controlled trial (RCT). Participants will be randomized to VR SCT (DiSCoVR) or VR relaxation training (VRelax; active control). DiSCoVR is a 16-session individual SCT, consisting of three modules: 1) emotion perception (recognizing facial emotions in a virtual shopping street); 2) social perception and theory of mind (observing social interactions between virtual characters and assessing their behavior, emotions and thoughts); and 3) application of higher-order social cognition in social interaction (role-playing personalized situations in VR). People receiving VRelax complete sixteen individual sessions, in which they receive psycho-education about stress, identify personal stressors, learn relaxation techniques, and explore relaxing immersive virtual environments.

Assessments will be performed at baseline, post-treatment, and 3-month follow-up. Primary outcomes are emotion perception (Ekman 60 Faces), social perception and theory of mind (The Awareness of Social Inference Test). Secondary outcomes include social functioning (Personal and Social Performance Scale), experiences and social interactions in daily life (experience sampling of emotions, social participation and subjective experience of social situations), psychiatric symptoms (e.g., depression, perceived stress, anxiety, positive and negative symptoms) and self-esteem.

**Discussion:**

To our knowledge, this will be the first RCT testing the efficacy of VR SCT. It will also investigate generalization to daily life social situations, the durability of treatment effects, and moderators and mediators of treatment success.

**Trial registration:**

On December 5, 2017, this trial was registered prospectively in the Dutch Trial Register as NTR6863.

**Electronic supplementary material:**

The online version of this article (10.1186/s12888-019-2250-0) contains supplementary material, which is available to authorized users.

## Background

For many people who have a psychotic disorder, it is a challenge to have meaningful daily activities, such as work, and to develop and maintain personal and romantic relationships; many of them experience problems in social functioning [1]. To function in daily life, it is necessary to adequately handle social situations and to be able to interpret the emotions, thoughts, intentions and behaviors of other people. These capabilities are generally referred to as *social cognition.* However, in people with a psychotic disorder, deficits in social cognition are common; a meta-analysis found large deficits in emotion perception and processing, social perception and theory of mind, compared to healthy controls [2]. Deficits in these key domains have been found to be important, proximal predictors for social functioning [3]. Social cognition has therefore become a target for psychosocial interventions aiming to improve social functioning through social cognitive training [4], a class of interventions also known as social cognition training (SCT).

SCT aims to improve social cognition, and ultimately social functioning through repeated practice with social stimuli (e.g., identifying emotions in pictures of faces), and compensatory strategy training (e.g., verbalization of salient facial features) [5]. A meta-analysis [6] of all types of SCT found large effects on emotion perception (recognition of emotions), and small to moderate effects on theory of mind (the ability to recognize and reflect on others’ mental states). A more recent meta-analysis [7], including only comprehensive forms of SCT (i.e., targeting multiple areas of social cognition), found a large effect on emotion perception and theory of mind, and a small to moderate effect on attribution style (judgments about the causes of events and others’ behavior). Together, these meta-analyses suggest that it is possible to improve social cognition with SCT in people with a psychotic disorder.

Some concerns have been raised, however, about the durability and generalizability of effects of SCT [4, 8]. While one meta-analysis [6] found a positive effect of SCT on community functioning, other reviews point out that treatment effects are most commonly and robustly found for lower-order social cognition processes such as emotion perception. On higher-order social cognition such as theory of mind, and social functioning, findings are more mixed and dependent on the specific measure used [4, 8]. We know from cognitive remediation research that integration with daily life is important for skills developed though cognitive training to generalize to social functioning [9]. Conventional SCT approaches may offer insufficient opportunity to practice in situations resembling real-life social interactions [8, 10]. Thus, effects of SCT on higher-order social cognitive processes and social functioning may be limited, as training materials may not sufficiently capture the complex, interactive and dynamic nature of real-life social situations.

The utilization of virtual reality (VR) for SCT has been proposed as a possible way to address this issue [4, 10]. VR is highly immersive, interactive and dynamic, and elicits psychological reactions similar to those in daily life [11]. Therefore, it is well-suited to simulate a variety of social situations and adequately reflect their complexity. At the same time, VR is controllable, which facilitates structured practice, and allows for situations to be scripted, repeated, varied, and personalized. Moreover, VR is a safe, accessible way to practice; participants know the virtual interactions can be stopped at any time, and that they can practice without negative repercussions and fear of embarrassment in their real social lives. Finally, VR is practical, as a wide range of situations can be generated quickly and practiced from one treatment setting, without traveling or accompanying participants in their daily environment.

VR is therefore a promising method to provide SCT. However, to our knowledge, no controlled studies of VR SCT for people with a psychotic disorder are available. Only one study on VR SCT has been published; a case report with two participants [12]. Improvements in social cognition and social functioning were reported after twenty sessions of a VR SCT. VR has also been used for social skills training (SST) in people with psychosis. A RCT of VR SST [13] among inpatients with schizophrenia (*n* = 91) showed that, compared to conventional SST, VR SST produced greater improvements in assertiveness and conversational skills, but no improvement was found in non-verbal skills. A pilot study of VR SST [14] reported improvement in social functioning, and VR measures revealed improvements in emotion perception, assertiveness, and time spent in conversations. Finally, pilot studies of VR SCT in people with autism spectrum disorder have reported improved emotion perception [15–17], attribution [16], theory of mind [15, 17], and (self-reported) social functioning [17]. Together, these studies suggest that VR SCT is promising.

To this end, we developed a novel VR SCT, Dynamic Interactive Social Cognition Training in Virtual Reality (DiSCoVR). In this randomized controlled trial (RCT), we aim to compare DiSCoVR to an active control condition, i.e., VR relaxation training (VRelax), in order to evaluate its efficacy on social cognition and social functioning. Moreover, we aim to assess the impact of the intervention on (the frequency and experience of) daily life social interactions, by using Experience Sampling Methodology (ESM), a diary method. We will also assess the durability of potential treatment effects in a 3-month follow-up period. Finally, we will perform moderator analysis to investigate who benefits from this VR SCT, and mediator analyses to examine variables mediating treatment effects. Thus, this study is the first RCT on VR SCT, using conventional measures of social cognition and functioning as well as experience sampling.

## Methods/design

### Participants

Participants are recruited from Dutch mental health treatment centers (University Center of Psychiatry of the University Medical Center Groningen, GGZ Drenthe, GGZ Delfland, and Zeeuwse Gronden). We recruit participants in two ways: 1) through advertisement of the study, by distributing posters and flyers, allowing participants to enroll themselves; and 2) through clinician referral: clinicians refer eligible and interested participants. Inclusion criteria are:

- A diagnosis of a psychotic disorder according to DSM-5 criteria, either determined by a structured clinical interview (e.g., Mini-international Neuropsychiatric Interview [18], Structured Clinical Interview for DSM [19], Schedules for Clinical Assessment in Neuropsychiatry [20]) in the past three years or verified using the MINI Plus at baseline.
- Deficits in social cognition, as indicated by a clinician.
- Age 18–65 years.

Exclusion criteria are:

- (Photosensitive) Epilepsy.
- An estimated IQ below 70, and/or a diagnosis of intellectual disability.
- Insufficient proficiency in the Dutch language.

Participants receive a compensation of €30 for each completed assessment, up to a total of €90. They also receive reimbursement for travel expenses incurred for the assessments.

### Design

**Fig. 1:**
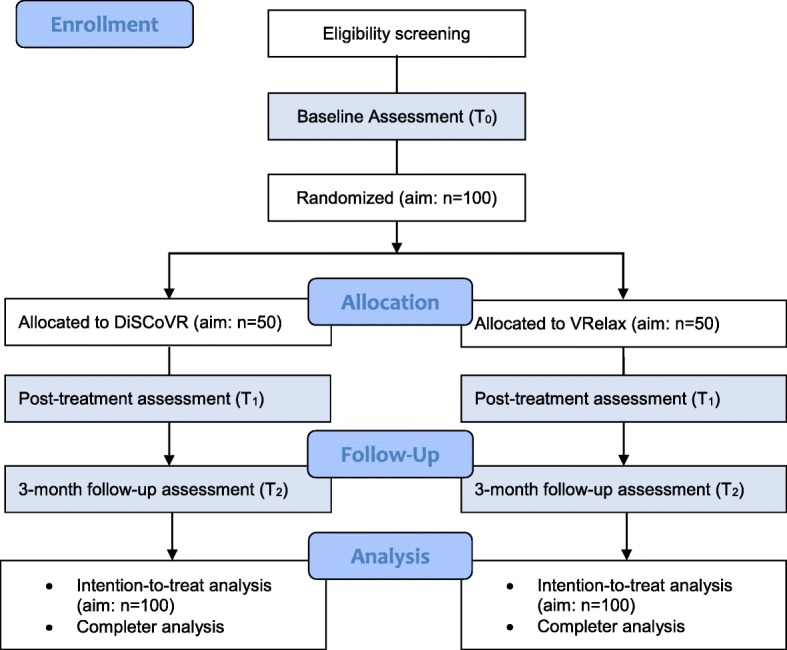
CONSORT flow diagram

The CONSORT inclusion flow diagram is shown in Fig. 1. This study is a multicenter single-blind randomized controlled trial (RCT) with two groups: 1) Dynamic Interactive Social Cognition Training in Virtual Reality (DiSCoVR), the experimental group; and 2) Virtual Reality Relaxation (VRelax), an active control group. Both groups receive treatment as usual alongside their designated intervention (DiSCoVR or VRelax). Participants complete three assessments: before the intervention (baseline, T_0_), directly after the intervention (post-treatment, T_1_) and three months after completing the intervention (follow-up, T_2_). After completion of the study, participants have the opportunity to receive the other treatment, if they wish.

### Interventions

#### DiSCoVR

DiSCoVR is a 16-session individual VR SCT, taking place twice a week over a period of eight weeks. DiSCoVR is provided by psychologists with (at minimum) a master’s degree in clinical psychology. A detailed treatment protocol describes the intervention, the software, the techniques that are used and the content of each session. Participants receive a workbook, containing session content summaries, exercises and worksheets (e.g., registration form of emotions they identified in their daily environment). DiSCoVR involves mass practice and compensatory strategy training. References to the patient’s personal situations and goals are made in examples and/or exercises whenever possible, to facilitate generalization of training material to daily life. To the same end, through homework exercises, participants are encouraged to practice with techniques and strategies they have learned in the intervention in daily life. These homework exercises are discussed at the start of each session. The intervention consists of three parts, structured to build upon each other in increasing complexity:

- Module 1: Emotion perception (5 sessions, 1–5). Participants explore a virtual shopping street where they identify emotions on virtual characters’ faces. Several characteristics (e.g., intensity of emotions, time to respond) can be altered to adjust the difficulty level to the training needs of the participant. Participants practice with several strategies (e.g., mimicking emotions) to aid in emotion recognition, which they are encouraged to try at home.
- Module 2: Social perception & theory of mind (4 sessions, 6–9). Participants view scripted social scenarios in VR, about which open-ended questions regarding behavior, emotions and thoughts are asked. These questions aim to stimulate mentalization. Participants can respond by stating their answer out loud. If they answer incorrectly, characters will give more explicit hints about their mental state, with intensified emotional expression. Participants continue to apply social strategies to understand situations (e.g., remembering how you felt in a similar situation), by choosing a strategy before practicing in VR. Participants learn about the connection between thoughts, emotions and behavior, and practice with recognizing these in VR scenarios, themselves, and in others around them.
- Module 3: Social interaction (7 sessions, 10–16). Participants apply social cognitive skills in VR roleplay exercises with the therapist. They can practice with situations they struggle with, or that help them to achieve the goals they have set for the intervention. The protocol provides sample roleplay exercises, targeted at various social cognitive skills (e.g., detection of sarcasm and hints). Outside of VR, participants continue to practice with identifying emotions, thoughts and behavior and understanding their interrelatedness. They also learn a social problem-solving technique, in which they 1) consider their own thoughts, emotions and behavior; 2) consider the other person’s thoughts, emotions and behavior; 3) consider different possible ways to react to a situation; and 4) role-play the different reactions and choose the most appropriate reaction to carry out in real life. Participants continue to practice using strategies (e.g., watching someone’s emotional response to you to determine whether you should talk to them), both in VR and in real life.

**Fig. 2:**
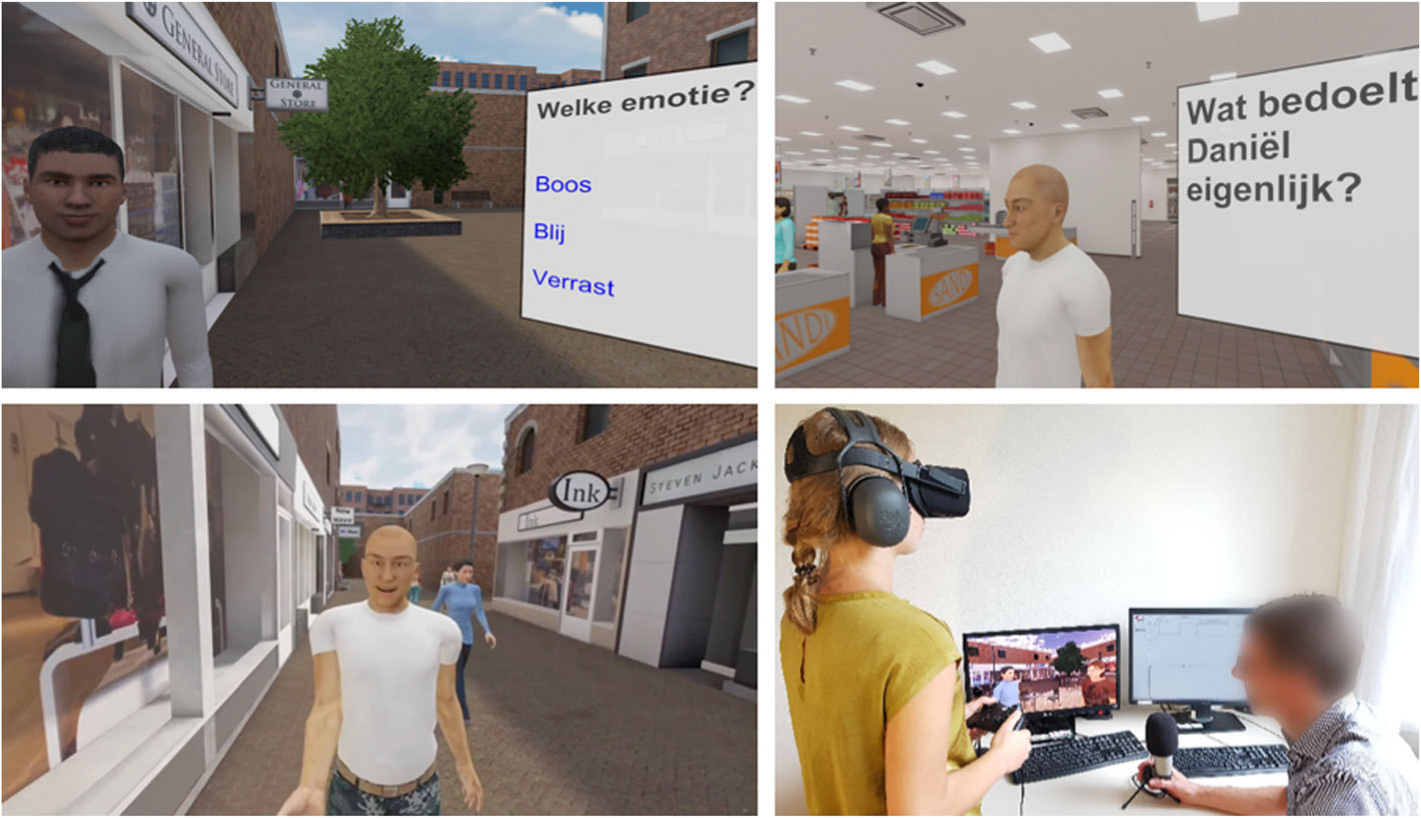
Pictures of DiSCoVR environments and set-up. Legend: Top left: module 1 (translation: which emotion? Angry, happy, surprised); top right: module 2 (translation: what does Daniël actually mean?); bottom left: module 3, as seen from perspective of participant; bottom right: module 3, interactive role play setup. The virtual environments are property of CleVR BV; images have been used with permission from CleVR BV

The immersive VR worlds (a shopping street, a super market, an office and a bar), developed by CleVR BV, are displayed with an Oculus Rift headset (Consumer Version 1). They are shown in Fig. 2. Participants explore and interact with the virtual environment using a joystick controller. For the role play exercises, voice transformation is applied to mask the therapist’s real voice. During the role plays, therapists can click buttons on the computer interface to trigger emotions and/or gesture animations (e.g., shrugging, pointing) in their virtual character.

#### VRelax

As an active control group, we chose VRelax, a VR relaxation therapy, as it matched important nonspecific characteristics of DiSCoVR (e.g., the use of VR, individual contact with a therapist twice a week), but did not target sociocognitive processes and therefore was not expected to improve social cognition. VRelax consists of sixteen sessions, provided twice a week in a period of eight weeks (the same as DiSCoVR), and aims to reduce both momentary (during sessions) and daily life stress and anxiety. VRelax is provided by therapists who either have 1) clinical experience in treating people with psychosis (e.g., psychiatric nurses); or 2) at least a bachelor’s degree in psychology. In VRelax, participants’ stressors are explored (i.e., which situations are stressful, and what are signs of stress). Participants learn to recognize stressful thoughts and rumination, and to apply coping strategies to address these (e.g., solving problems, asking for support and comfort, finding distraction). They also practice with relaxation exercises, such as breathing exercises, a body scan, grounding, and progressive muscle relaxation. Participants are encouraged to practice these techniques at home.

**Fig. 3:**
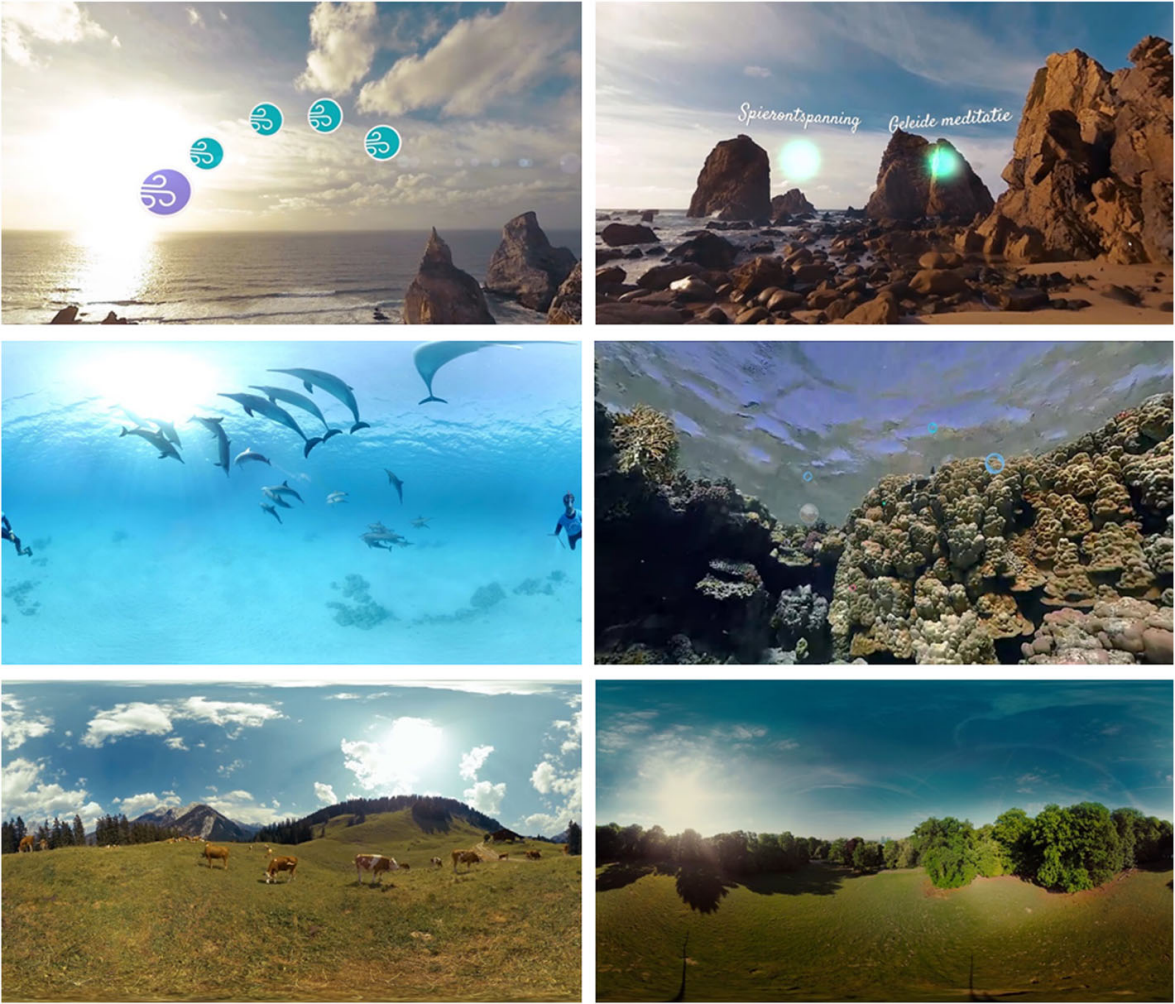
VRelax environments, including relaxation exercises. The virtual environments are property of Viemr BV; images have been used with permission from Viemr BV

Each session starts with a discussion of stressors and negative emotions in the past few days. In some sessions, this is followed by psycho-education (e.g., about stress, coping and rumination). A large portion (±25 min) of each session is reserved for relaxation in VR. Immersive 360° video clips are shown with a Samsung Gear VR headset, running VRelax software developed by Viemr BV. Participants can visit a range of relaxing environments (Fig. 3), such as a sea in which they can swim with dolphins, a coral reef, a beach and a mountain meadow. They can control the environment using gaze; for example, to switch between environments or to engage with interactive elements (e.g., popping air bubbles underwater). Meditation exercises are embedded in the VR environment (e.g., guided meditation). Finally, part of each session is reserved for (learning to apply) relaxation techniques. These can be skipped if participants prefer to spend more time in VR.

### Materials and measurement instruments

The measures used in this study are summarized in Table 1.

**Table 1:** Measures used in this study

Domain	Instrument	Method	Baseline (T_0_)	Post-treatment (T_1_)	Follow-up (T_2_)
*Primary outcome measures*					
Emotion Perception	Ekman 60 Faces (FEEST)	Picture task	x	x	x
Social Perception / ToM	TASIT-III	Video task	x	x	x
*Secondary outcome measures*					
Social functioning in daily life	Questions about social interactions, activities and momentary mental states	ESM questionnaire	x	x	x
Social functioning	PSP	Interview	x	x	x
Psychotic Symptoms	PANSS	Semi-structured interview	x	x	x
Social Anxiety	SIAS	Questionnaire	x	x	x
Paranoid Thoughts	GPTS	Questionnaire	x	x	x
Depression	BDI	Questionnaire	x	x	x
Anxiety	BAI	Questionnaire	x	x	x
Stress	PSS	Questionnaire	x	x	x
Self-esteem	SERS	Questionnaire	x	x	x
Information processing	TMT	Task	x	x	x
Demographic and clinical background	Questions	Interview	x	x	x
*Baseline diagnostic measures*					
Premorbid intelligence	NLV	Task	x		
Diagnostic	M.I.N.I. Plus	Structured Interview	x		
*Measures during sessions*			Session(s)		
Cyber sickness	SSQ	Questionnaire	S3		
Emotions	Questions	Questionnaire	VRelax: each session (2x)		
Performance on VR task	Social cognition tasks in VR	Task	DiSCoVR: S1–9		
Behavior in VR	Reaction time, interpersonal distance	Task	DiSCoVR: S1–9		

#### Primary outcome

##### Facial affect recognition

Ekman 60 Faces, Facial Expression of Emotions Stimuli and Tests (FEEST) [21]. 60-item computerized picture test assessing facial affect recognition. Participants view pictures of six emotions (happy, sad, angry, disgusted, surprised, anxious) and identify the expressed emotion. Total scores (0–60) are used for analysis.

##### Social perception and theory of mind

The Awareness of Social Inference Test part III (TASIT, Dutch version) [22, 23]. Short video clips showing social situations containing lies and sarcasm. Participants answer four questions after each video about the intentions, intended messages, beliefs and emotions of the characters. Total scores (0–32) are used for analysis. Parallel versions are used; the administration order (A-B-A or B-A-B) is randomized across participants.

#### Secondary outcomes

##### Social functioning

Personal and Social Performance Scale (PSP) [24]. An interview assessing the degree of disability (from 1, “absent”, to 5, “severe”) in four areas of social functioning: 1) socially meaningful activities (e.g., work, study); 2) personal and social relationships (e.g., friendships); 3) self-care (e.g., hygiene); and 4) disturbing and aggressive behavior (e.g., problems with law enforcement). The ratings for each domain are combined into one score from 0 (very low) to 100 (very high), which will be used for analysis.

##### Momentary mental states and social functioning in daily life

Experience Sampling Method (ESM) dairies are used to study the effects of the interventions on daily life interactions. An online questionnaire (21–33 items, included in Additional file 1: Table S1) is administered ten times a day for seven days at semi-random moments, by sending participants text messages containing a link to the questionnaire, which they can complete in the internet browser on a smartphone. Participants use their own smartphone or one provided by the research team. The ESM questionnaire assesses: 1) emotional state (positive and negative affect and stress level; thirteen items), on a visual analogue scale (VAS; 0–100); 2) current activities and activities since the last questionnaire (five items, to assess social and societal participation), selected from a multiple choice menu, and a VAS (0–100) on the enjoyability of the activity; 3) current social interactions and interactions since the last questionnaire (fourteen items), including the enjoyability of the interaction, feelings of being accepted, knowing how to respond, and feeling able to tell what the other person is feeling (VAS, 0–100); and 4) taking social initiative (two items; VAS, 0–100).

##### Psychiatric symptoms

The following measures of psychopathology will be administered, since (social) cognitive training may improve psychiatric symptoms, and symptom levels could be a moderator or mediator of treatment effect:

- The Positive and Negative Syndrome Scale (PANSS) [25], a semi-structured interview assessing positive (7 items, range 7–49), negative (7 items, range 7–49) and general (16 items, range 16–112) symptoms of psychosis in the past week on a 7-point scale (from 1, “absent”, to 7, “very severe”). We will analyze the three subscales separately as well as the total score.
- The Social Interaction Anxiety Scale (SIAS) [26], a 20-item self-report questionnaire measuring verbal and non-verbal social anxiety on a 5-point Likert scale. We will analyze the total score.
- The Green Paranoid Thought Scale (GPTS) [27], a self-report questionnaire measuring paranoid thoughts of social reference ([16] items, part A) and social persecution (16 items, part B) in the past month on a 5-point Likert scale. We will analyze both subscales separately.
- Perceived Stress Scale (PSS) [28], a ten-item self-report questionnaire on symptoms of stress in the past week, indicated on a 5-point Likert scale. We will analyze the total score.

#### Other

##### Self-esteem

The Self-Esteem Rating Scale (SERS) [29]. The SERS is a twenty-item self-report questionnaire using a 7-point Likert scale, assessing feelings of self-worth and competence. Total scores will be analyzed.

##### Information processing and executive functioning

The Trailmaking Test (TMT) [30]. In TMT-A, participants connect numbers in circles scattered across a page in consecutive order; in TMT-B, participants connect numbers and letters in circles scattered across a page, alternating between the letters and circles in consecutive order (1-A-2-B, etc.). The completion time in seconds for both parts is analyzed.

##### Demographic and clinical background

Questions on different clinical and demographic variables, including: age, gender, handedness, eyesight, family history of psychiatric illness, education level of participant and participant’s parents, work history, diagnosis and illness duration (administered only at baseline); and housing status, employment status, substance use, medication use, hospitalizations and psychotic episodes (administered at each assessment).

##### Premorbid intelligence

The Dutch version (Nederlandse Leestest voor Volwassenen, NLV) [31] of the National Adult Reading Test [32]. Fifty increasingly uncommon words are read aloud by participants. Correct pronunciations are awarded a score of two points. The total score is used for analysis. The NLV is only administered at baseline.

##### Eligibility check

Mini International Neuropsychiatric Interview Plus (M.I.N.I.-plus) [18]. A structured clinical interview on symptoms of psychosis, depression and mania. The MINI-plus is administered at baseline to verify a diagnosis of psychotic disorder, if a diagnosis has not been determined by a structured clinical interview in the past three years.

#### Measures during treatment sessions

##### Cyber sickness

Simulator Sickness Questionnaire (SSQ) [33]. A self-report questionnaire on 27 common symptoms of simulator sickness (e.g., headache, nausea, dizziness), which will be administered right after the VR session 3 of both interventions to evaluate negative side effects of using VR. We will use the total score for analysis.

##### Performance and behavior during VR tasks

In the DiSCoVR group, the VR program will log several parameters during practice sessions (e.g., the avatars encountered, answers given to questions, time taken to answer, the number of answer attempts and interpersonal distance).

##### Emotions before and after relaxation

In the VRelax group, before and after each session in VR, participants complete a short questionnaire in which they rate their emotions (relaxed, anxious, gloomy, tranquil, stressed, excited, nervous and content) on a 0–100 VAS.

#### Assessment

The assessments are carried out by trained, independent research assistants. This study is single-blind, meaning that research assistants do not know to which intervention participants have been assigned. This is achieved by instructing coordinators, therapists and participants not to divulge group allocation to research assistants. Other precautions include storing data revealing group allocation (e.g., therapist worksheets) in a separate location and using different assistants for each measurement as much as possible. Blinding is evaluated with a self-report form for research assistants at the end of the post-treatment and follow-up assessments. We will perform a sensitivity analysis by testing the treatment effect only for measurements where research assistants reported being completely blinded to group allocation.

#### Randomization procedure

We utilized permuted block randomization with randomized block sizes (2, 4 or 6) using the R package ‘blockrand’ [34]. For each participating treatment center, we created strata for age (ten-year increments) and gender (i.e., ten strata per center). This resulted in a master randomization list with either ‘A’ or ‘B’ in each stratum. An independent employee then added an additional randomization by assigning DiSCoVR or VRelax to either A or B in the master randomization list using coin flips, once for each treatment center. Upon completing T_0_, the participants will be placed in the appropriate stratum in order of inclusion by the independent randomizer, determining their group assignment.

#### Protocol fidelity

Therapists in both conditions will complete an eight-hour training. Monthly group supervision, in which all ongoing cases are discussed, will be provided for both conditions, to aid therapists and improve protocol fidelity. Therapists who are unavailable for these meetings will receive individual supervision. A weekly consultation session with the research team will also be available. After each session, therapists will complete a session form, on which they indicate whether they completed all parts of the protocol for that session, and whether any anomalies occurred.

#### Drop-out

Participants will be able to discontinue the study at any point. They can also refuse participation in part of the study, such as the ESM questionnaires. For sensitivity analyses, participants will be considered treatment completers if they complete twelve sessions or more (> 75%). If participants drop out of treatment, they are asked to continue the assessments. If participants refuse or miss T_1_, they are still contacted for T_2_.

#### Data management

Participant data will be coded using a study ID. Personal information and informed consent will be stored separately to ensure anonymity. We will use an electronic case report form; paper source data will be stored securely at each respective location. An independent monitor, who performs yearly routine inspections, will be appointed to ensure study quality and integrity.

#### Analysis

For our primary outcome variables (theory of mind and emotion perception), previous meta-analyses [6, 7] have found effect sizes ranging between moderate (*d =* .46, theory of mind) and large (*d* = .84, emotion perception). We therefore assumed a conservative effect size of .5. With β = .80, α = .05, two groups and three assessments, we found we would need 86 participants. Assuming a drop-out rate of 13% [35], we concluded that the sample should consist of 100 participants (50 in each group).

The groups will be compared at baseline using t-tests for normally distributed variables, and Mann-Whitney U-test or χ^2^ test for other variables. Variables with a significant baseline difference between the groups will be added to the analysis as a covariate.

We will use multilevel mixed-model linear regression analysis in accordance with the intention to treat principle, with repeated assessments (level 1) nested within participants (level 2). Time and group and their interaction term will be included as fixed effects. Treatment effects will be evaluated by examining the interaction between time and group. A random intercept will be included for the participant level to account for the repeated measures. A random slope will be included for time if this improves the model, according to the AIC value. The post-treatment and follow-up assessments will be analyzed separately, comparing each to the baseline. We will also perform a multilevel regression analysis on treatment completers, defined as people who have completed at least twelve sessions (75%) of their allocated intervention. Furthermore, moderator (e.g., clinical and demographic variables) and mediator analyses will also be conducted. Significance will be accepted at α = .05 (two-tailed); in case of multiple measurements within the same domain, a correction will be applied to α.

#### Procedure

Clinicians will approach potential participants and gauge their interest in the study. If patients express interest, or if potential participants approach the research team directly, they will be contacted and screened by the research team. They will receive written information about the study and will be given a one-week consideration period. If they are still interested after this period, participants will provide written informed consent, collected by the research assistant, and the baseline assessment will take place. In the week after the first assessment, the baseline ESM questionnaire period will take place. After this, participants are randomized to either DiSCoVR or VRelax, and receive sixteen DiSCoVR or VRelax sessions. When treatment has been completed, the post-treatment assessment will take place, followed by another ESM period of one week. Finally, three months after completing treatment, participants will complete the last follow-up assessment, followed by the final ESM questionnaire week.

## Discussion

The main goal of this trial is to evaluate the effect of DiSCoVR on social cognition and social functioning. We hypothesize that the DiSCoVR group will show greater improvements in emotion perception and theory of mind than VRelax at post-treatment, and that this difference will be maintained at 3-month follow-up. Additionally, we expect that participants in the DiSCoVR group will experience greater improvement in social functioning, as measured by an interview (PSP). Furthermore, we hypothesize that people in the DiSCoVR group will show more improvement in daily-life social functioning (i.e., feelings of acceptance, subjective social cognition, social participation and social initiative) than people who receive VRelax. Since VRelax targets stress, anxiety, coping and stress reactivity, we expect that participants in the control group will show equal or larger improvements in perceived stress and general anxiety.

Very little is currently known about mediators and moderators of treatment effects of SCT [4]. Therefore, mediation and moderation analyses will be explorative in nature. A meta-analysis on SCT [6] found that illness duration and education level were moderators of treatment effects on social cognition. We therefore postulate that these variables may moderate the treatment outcome of DiSCoVR, but we will also investigate other moderators (e.g., baseline levels of social anxiety and paranoia).

However, no meta-analysis thus far has investigated mediators of SCT efficacy. Both Eack et al. [36] and Hogarty et al. [37] found that improvements in neurocognition mediated improvements in social functioning after Cognitive Enhancement Training, a form of SCT targeting both social cognition and neurocognition. Neurocognition may therefore be a mediator of treatment outcome in the present study. However, since little is currently known about mediators of SCT treatment effects, we will explore additional variables.

While SCT is not a new method, the present study intends to improve upon existing methods by allowing participants to apply social cognition in personalized virtual environments and situations resembling daily life. This trial will also address some of the gaps in knowledge about SCT [4], particularly concerning generalization to daily life and durability, but also moderators and mediators of training effects.

Since there are presently no RCTs on VR SCT, the present study will shed light on the efficacy of this innovative method. If it proves to be effective, it could be an important tool to improve social cognition and social functioning – which is sorely needed, since durable functional recovery after psychosis is still uncommon [38]. Finally, because social cognitive deficits are a transdiagnostic characteristic [39], VR SCT may be a useful tool to remediate problems in social cognition and social functioning across a range of psychiatric populations, such as autism spectrum disorders.

## Additional file

Additional file 1: **Table S1.** ESM questionnaire. (DOCX 21 kb)

## Data Availability

The datasets used and/or analyzed during the current study will be available from the corresponding author on reasonable request.

## Abbreviations

BAI: Beck Anxiety Inventory
BDI: Beck Depression Inventory
DiSCoVR: Dynamic Interactive Social Cognition Training in Virtual Reality
ESM: Experience Sampling Method(ology)
FEEST: Facial Expressions of Emotion: Stimuli and Tests
GPTS: Green Paranoid Thought Scale
M.I.N.I.-plus: Mini International Neuropsychiatric Interview Plus
NART: National Adult Reading Test
NLV: Nederlandse Leestest voor Volwassenen
PANSS: Positive and Negative Syndrome Scale
PSP: Personal and Social Performance Scale
PSS: Perceived Stress Scale
RCT: Randomized Controlled Trail
S: Session
SCT: Social Cognition Training
SERS: Self-Esteem Rating Scale
SIAS: Social Interaction Anxiety Scale
SSQ: Simulator Sickness Questionnaire
T_0_: Baseline
T_1_: Post-treatment
T_2_: Follow-up
TASIT: The Awareness of Social Inference Test
TMT: Trailmaking Test
UMCG: University Medical Center Groningen
VR: Virtual Reality
VRelax: Virtual Reality Relaxation
