# Supplementary material for: Dynamic Interactive Social Cognition Training in Virtual Reality (DiSCoVR) for social cognition and social functioning in people with a psychotic disorder: study protocol for a multicenter randomized controlled trial
Source: BMC Psychiatry. 2019 Sep 5;19:272. doi: 10.1186/s12888-019-2250-0 (PMC6727396; doi:10.1186/s12888-019-2250-0)
Supplement: Supplementary file 1 — Table S1. ESM questionnaire. (DOCX 21 kb) [file 12888_2019_2250_MOESM1_ESM.docx]

Additional file 1: Table S1 ESM questionnaire.

| Question | Answer option(s) |
| --- | --- |
| I’m feeling cheerful | VAS 0-100 |
| I’m feeling anxious | VAS 0-100 |
| I’m feeling content | VAS 0-100 |
| I’m feeling irritated | VAS 0-100 |
| I’m feeling nervous | VAS 0-100 |
| I’m feeling stressed | VAS 0-100 |
| I’m feeling gloomy | VAS 0-100 |
| I’m feeling insecure | VAS 0-100 |
| I’m feeling enthusiastic | VAS 0-100 |
| I’m feeling relaxed | VAS 0-100 |
| I’m feeling suspicious | VAS 0-100 |
| I’m feeling safe | VAS 0-100 |
| Where are you right now? | At home, at school, at work, with family, with friends/acquaintances, at a bar/restaurant/library, at the supermarket/store, in nature, with a clinician, traveling, elsewhere. |
| What are you doing right now? | Working/studying, exercising/walking/cycling, hobby (e.g., making music, carpenting), activity (e.g., shopping, concert), watching TV/Youtube/Browsing the internet, gaming, texting, talking to someone, something intimate (e.g,, cuddling), eating, housekeeping/groceries/administrative, self-care (e.g., showering, doing make-up, shaving), taking care of someone else (e.g., child or parent), sleeping, resting/nothing, traveling, something else. |
| How enjoyable do I find this activity? | VAS 0-100 |
| What was your most important activity since the last measurement? | Working/studying, exercising/walking/cycling, hobby (e.g., making music, carpenting), activity (e.g., shopping, concert), watching TV/Youtube/Browsing the internet, gaming, texting, talking to someone, something intimate (e.g,, cuddling), eating, housekeeping/groceries/administrative, self-care (e.g., showering, doing make-up, shaving), taking care of someone else (e.g., child or parent), sleeping, resting/nothing, traveling, something else. |
| How enjoyable did you find this activity? | VAS 0-100 |
| Who are you with right now? | Nobody (alone), my partner, my child(ren), father/mother/brother/sister (who live with me), housemate (not family), family member (who does not live with me), friend/acquaintance, coworker/classmate, clinician, stranger(s) |
| If not alone: I feel accepted by this person | VAS 0-100 |
| If not alone: I feel insecure with this person | VAS 0-100 |
| If not alone: I feel connected with this person | VAS 0-100 |
| If not alone: I enjoy this company | VAS 0-100 |
| If not alone: I know how to react to this person | VAS 0-100 |
| If not alone: I understand how this person is feeling right now. | VAS 0-100 |
| Since the last measurement, I was also with... | Nobody (alone), my partner, my child(ren), father/mother/brother/sister (who live with me), housemate (not family), family member (who does not live with me), friend/acquaintance, coworker/classmate, clinician, stranger(s) |
| If not alone: I felt accepted by this person | VAS 0-100 |
| If not alone: I felt insecure with this person | VAS 0-100 |
| If not alone: I felt connected with this person | VAS 0-100 |
| If not alone: I enjoyed this company | VAS 0-100 |
| If not alone: I knew how to react to this person | VAS 0-100 |
| If not alone: I understood how this person was feeling. | VAS 0-100 |
| Since the last measurement I have initiated social contact | VAS 0-100 |
| In the time since the last measurement I would have preferred more company | VAS 0-100 |
